# Comparative Analysis of Male Cyclist Population in Four Asia Countries for Anthropometric Measurements

**DOI:** 10.3390/ijerph191610078

**Published:** 2022-08-15

**Authors:** Arunachalam Muthiah, Yu-Chi Lee

**Affiliations:** 1 School of Industrial Design, Karanavathi University, Gandhinagar 342422, Gujarat, India; 2 College of Management and Design, Ming Chi University of Technology, New Taipei City 243303, Taiwan

**Keywords:** cycling, ethnicity, anthropometry, design, ergonomics

## Abstract

This study aimed to compare the anthropometric variables of male cyclist samples from India, China, Singapore, and Taiwan. The cyclist’s body dimensions were measured among 413 randomly chosen males (aged between 18 to 60), which included 104 Indians, 106 Taiwanese, 100 Singaporeans, and 103 Chinese. Based on the previous research articles, the considered 17 anthropometric variables were weight, stature, BMI, buttock extension, shoulder height (sitting), shoulder-elbow length, elbow height (sitting), lower leg length, knee height, acromion-grip length, hand length, elbow-hand length, buttock-popliteal length, buttock-knee length, elbow-to-elbow breadth, hip breadth (sitting), and foot breadth. Using statistical techniques (descriptive statistics, the Mann–Whitney U test, and Kruskal–Wallis H test), the data were analysed in SPSS, version 25.0. The results of the statistical analyses showed significant differences among the cyclists across selected anthropometric characteristics, except for the weight and sitting-related anthropometric measurements. The outcome of the descriptive statistics (percentile values), such as the percentile range (5th to 95th percentile), could be applied to the seat-height adjustment system to cover 95% of the bicyclist population. These types of implantation could enhance the ergonomic benefits for the bicyclist.

## 1. Introduction

The World Health Organization (WHO) declared that people engaged in physical activity have a significant affirmative effect on health development, such as walking and cycling [1]. The number of recreational cyclists is increasing in North America [2,3], Canada [4], various European countries [5], and also Asian countries [6]. In recent years, with the improvement of road construction and the promotion of healthy lifestyles in Asian countries, more and more people have begun to participate in cycling activities [7]. For example, the number of cyclists in Taiwan has tripled in just three years, and 80% of them are for recreational exercise purposes, as reported by the Council for Economic Planning and Development of Taiwan. Among other Asian countries (e.g., China, India, and Singapore), the overall cycling rates are continuously increasing [8]. This means an incredible demand for bicycle use is occurring. Moreover, an improper bicycle design and body fitting might cause discomfort, neck pain, muscle fatigue, and poor riding performance due to the awkward postures used [9,10,11]. Hence improving a cyclist’s riding comfort through designs with ergonomic concepts became vital. One of the critical features influencing the riding posture and performance was reported as anthropometric data [12], such as body-surface dimensions and frontal-body dimensions [13]. Many studies have recommended that anthropometric data can achieve better human–machine interactions, comfort, and performance, especially in cycling, bike design, and bike fitting [14,15,16,17].

Anthropometry has been indicated as an indispensable reference for product design to enhance the user’s performance and comfort. Thus, many whole-body anthropometric databases have been reported in various Asian countries, for example, Taiwan [18], the Philippines [19], Thailand [20], Turkey [21], Bangladesh [22], Iran [23], Malaysia [24], Western India [25], Indonesia [26], Singapore [27], and India [28]. The anthropometric differences among countries may be attributed to racial, social, and economic environments [29]. Regrettably, some previous studies are outdated and need to be updated.

With the changes in living habits and economic development, the human body’s dimensions also gradually change [27,30,31]. Thus, the difference in anthropometric data between various nations and the regional population is observed [29,32]. This means the anthropometric data are population-specific and difficult to use for different ethnic populations [23,24]. Therefore, exploring the differences in anthropometric data among different ethnicities or countries is valuable. Some studies have investigated the ethnic differences of body dimensions in the same country. For example, Widyanti et al. [33] and Hartono [26] measured Indonesian anthropometry data under different ethnicities (e.g., Minangkabau, Javanese, Sundanese, Drills, Chinese, and Non-Chinese). Moreover, Bhattacharjya and Kakoty [34] reported the ethnic differences in anthropometric data obtained from the Boro, Garo, Hira, Karbi, and Rabha living in India. Moreover, the same race coming from different countries might exhibit a dissimilarity in body dimensions. Lin et al. [29] first conducted a study to compare the anthropometric characteristics among four East Asian populations (the Taiwanese, Chinese, Japanese, and Koreans). Subsequently, Sadeghi et al. [23] compared the anthropometric data of Iranians with the previous Asian countries. Moreover, Chuan et al. [32] investigated the differences in anthropometric data between the Singaporean and Indonesian populations. Da Silva et al. [35] completed a comparison of anthropometry of Brazilian and US military populations and applied the results to a flight deck design. Rahman et al. [36] collected the anthropometric measurements of Malaysians and compared them to Indonesians, Filipinos, and Thai populations on sitting and standing body dimensions. All the mentioned studies showed that differences in ethnicity and countries were found in the anthropometric data.

However, due to the original human anthropometric data in different countries being measured by different research groups, most previous studies could only use the mean of the body dimensions and bodily proportions as parameters for comparing the body dimensions between different populations. This kind of comparison could only provide indirect evidence of the population differences in anthropometric data and the lack of statistical significance tests to support the fact. This may cause errors in practical applications. Hence, the current study was to conduct a cross-nation anthropometric data collection in Taiwan, Singapore, India, and China, and to compare the differences in the various body dimensions related to cycling design among the four Asian populations. The aim was divided into two objectives for better achievement as follows: (1) To present the descriptive analysis of Asian bicyclists’ anthropometric variables for bicycle design; (2) to compare the anthropometric variables of Asian bicyclist samples by using statistical techniques. The findings of this study can provide useful information for the ergonomics consideration of different countries during the bicycle design process. Further, it would be used in a CAD environment for the virtual ergonomics assessment of bicycles. In addition, the data could be processed further with statistical packages for developing a boundary-human model (virtual manikins) during the virtual ergonomics assessment of bicycles.

## 2. Materials and Methods

### 2.1. Participants

Since the bicyclist population of Asian countries (Singapore, Taiwan, India, and China) is still unknown, the minimum sample size was calculated (*n* ≥ 385) using the following parameters and Equation (1) [37]. Where the confidence level = 95% (*Z* = 1.96); with a sample proportion of 50% (*p* = 0.5) and the margin of error is 5% (*e* = ±0.05).

(1) $$n=\frac{Z^{2}p\left(1-p\right)}{e^{2}}$$

The cyclists’ body dimensions were measured among 413 randomly chosen males (aged between 18 to 60), which included 104 Indians, 106 Taiwanese, 100 Singaporeans, and 103 Chinese. These surveys were conducted in Singapore (Nanyang Technological University), Taiwan (Ming Chi University of Technology), China (South China University of Technology), and India (Karnavati University). Most of the subjects (bicyclists) were students or employees of these universities. We assumed that all the subjects of their individual countries were representatives of the bicyclist population with good health. The subjects with previous health issues (such as motor skills, bone fractures, etc.) were disallowed in the survey. All subjects were provided with a consent document for measuring their anthropometrics with an understanding of the research purpose. Due to a lack of manpower, the gender of investigators (all men), and time constraints, this study mainly measured the body dimensions of male samples.

### 2.2. Selection of Anthropometric Variables

**Figure 1:**
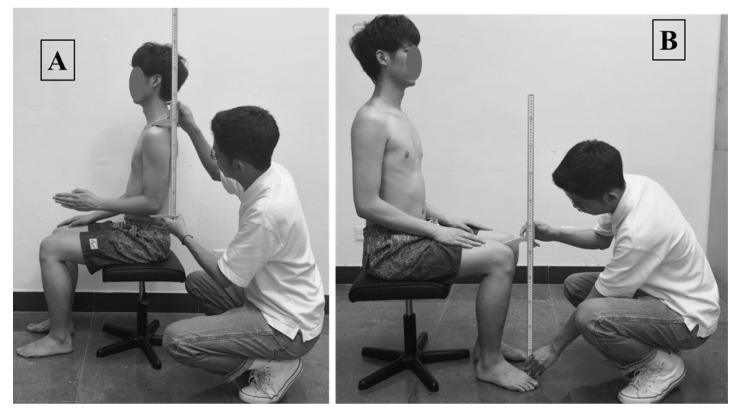
Sample picture captured during measurements. Note: (**A**) shoulder-elbow length; (**B**) knee height.

We measured 17 anthropometrics (which included stature body height, buttock extension, shoulder height (sitting), shoulder-elbow length, elbow height (sitting), lower leg length, knee height, acromion-grip length, hand length, elbow-hand length, buttock-popliteal length, buttock-knee length, elbow-to-elbow breadth, hip breadth (sitting), foot breadth, BMI, and weight). These measurements were recognized from earlier research articles [14,28,38,39,40,41,42,43], which investigated/studied affairs related to ergonomics in bicycle/two-wheeler designs. According to the ISO 7250-1: 2008(E) standards, the anthropometric measurement procedures were followed to obtain the bicyclists’ body dimensions (see Figure 1).

### 2.3. Measuring Instruments

**Figure 2:**
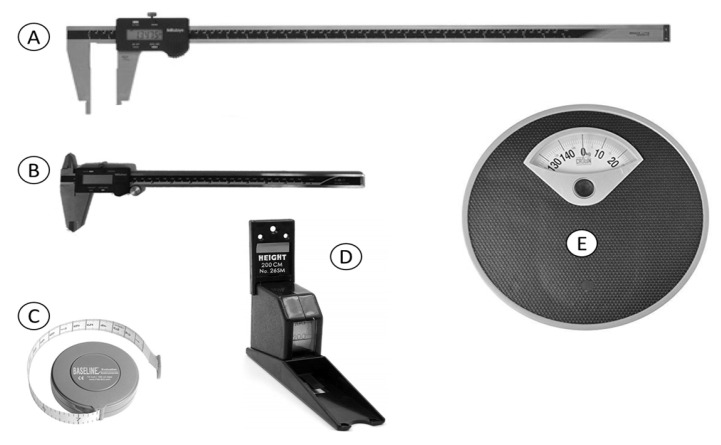
Measuring instruments used in the survey. Note: (**A**) larger sliding caliper; (**B**) small sliding caliper; (**C**) plastic measuring tape; (**D**) stadiometer and (**E**) weighing scale.

Manual anthropometric measuring apparatus and equipment were used due to the reason of accuracy/preciseness, ease of portability, and affordability [44]. Each piece of equipment was calibrated before obtaining the anthropometric measurements of bicyclists. In total, five pieces of equipment (see Figure 2) were used during the data collection process. One larger sliding caliper (make: Mitutoyo Corporation: Kawasaki, Japan; range: 0–700 mm; accuracy: 0.02 mm; resolution: 0.01 mm) was used to measure the length/height/width of body segments. A small sliding caliper (make: Mitutoyo Corporation- Kawasaki, Japan; 0–300 mm measurement range; 0.02 mm accuracy and 0.01 mm resolution), stadiometer-height measuring tape (Model: Gadget Hero, Beijing, China; Maximum 200 cm), nonstretchable plastic measuring tape (2000 mm), and portable weighing scale (138 kg maximum capacity, model: Crown Classic, New Delhi, India) were also used for collecting the anthropometric data.

### 2.4. Measurement Procedure

**Figure 3:**
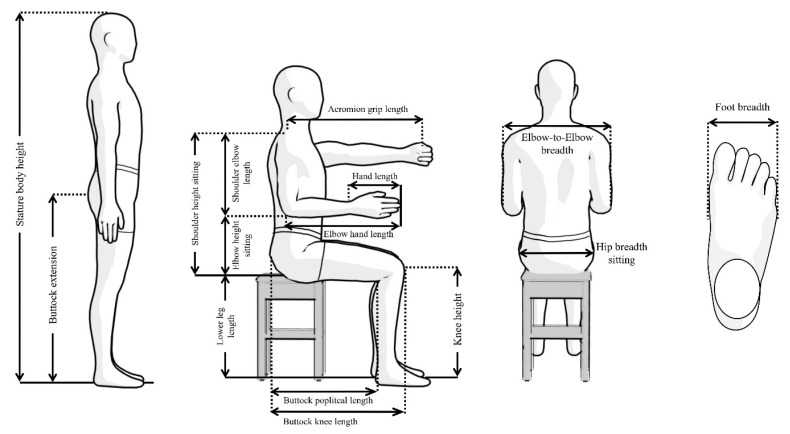
Anthropometric measurements.

Before the measurement procedure started, the participants were informed regarding the measurement procedures and protocols for the data collection. Additionally, the participants were asked to provide their written consent for the data collection, which was prepared according to the Helsinki guidelines and approved by the committees from the four mentioned universities. The 15 measurements (see Figure 3), BMI, and body weight were carefully observed by well-trained anthropometrist, who are familiar with anthropometry and human-body landmarks for error-free and reliable measurements. Weight, stature, and buttock extension were measured in the standing position of the participants. During these measurements, the participants were asked to stand in an anatomical position on a flat floor. Similarly, the other thirteen measurements (shoulder height (sitting), shoulder-elbow length, elbow height (sitting), lower leg length, knee height, acromion-grip length, hand length, elbow-hand length, buttock-popliteal length, buttock-knee-length, elbow-to-elbow breadth, hip breadth (sitting), and foot breadth) were observed in the sitting position with adjustable stoles. During these measurements, the participants were asked to keep their torso in an erect manner (with their shoulders and head aligned with the same vertical plane), their knees together without any gaps, and their feet on the flat floor. All the measurements were recorded in the participant’s semi-nude clothing condition. Since the intra-/inter- reliability assessment anthropometry results were highly reliable, the measurements observed in the datasheet from a single trial were only for future analysis.

### 2.5. Intra-/Inter- Reliability Assessment of Anthropometry

Before the anthropometric measurements were conducted on the cyclists of each country, inter-observer and intra-observer reliability tests were conducted on 10 randomly chosen healthy cyclists to assess the precision of the linear and mass measurements. To ensure the precision and accuracy in the measurement of all the anthropometric data, the reliability of anthropometry was estimated as the technical error of measurement (%TEM) of the inter-/intra-observer. This % TEM helped us to understand the manual or instrumental errors.

During the inter-reliability assessment, anthropometrists-1 and anthropometrists-2 measured the anthropometrics for 10 cyclists on the same day. For the intra-reliability assessment, anthropometrists-1 measured all the anthropometrics during the first week. In the subsequent week, the same anthropometry was followed by the anthropometrists-1 to estimate the %TEM of the intra-reliability assessment.

The %TEMs of the intra-/inter- were calculated in a spreadsheet using a set of %TEM equations, as stated in a previous research article [28]. In Appendix A, Table A4 presents the intra-/inter- reliability assessment of the anthropometrics with respect to countries. The %TEM of intra-reliability ranged from 0.15% to 1.73% across four countries. For the %TEM of inter-reliability, the estimation ranged from 0.11% to 1.57% across four countries.

### 2.6. Data Analysis

Using the IBM SPSS version 25.0 software (IBM: Armonk, NY, USA), the anthropometric data of the counties were analysed for the mean, standard deviation, maximum, minimum, range, and percentile distributions (5th, 50th, and 95th). Since the Kolmogorov–Smirnov test was used for *n* ≥ 50, the Shapiro–Wilk test was more appropriate for the small sample sizes (50 samples). However, it can also handle larger sample sizes [45]. The Shapiro–Wilk test was performed to assess the data’s normality at a confidence level of *p*-values of <0.05. Due to various reasons (such as limited samples, anthropometric variability, etc.), the normality test results imply that the data were not normally distributed. Henceforth, the differences among the four Asian countries were determined using the non-parametric Kruskal–Wallis test for all anthropometric measurements. Moreover, non-parametric statistics analyses (i.e., Mann–Whitney U Test) were performed to understand the difference between every two countries’ cyclists’ body dimensions. The comparison was performed in the following manner: Singapore (SGD) vs. Taiwan (ROC); SGD vs. China (PRC); SGD vs. India (INR); ROC vs. PRC; INR vs. ROC; INR vs. PRC. The basic cyclist characteristics were calculated by the following equation and methods. The body-surface area was estimated based on the Fujimoto and Watanabe formula [46]. As per Nes et al. [47], the HUNT equation (HRmax (beats/min) = [211 − 0.64 × Age]) is the slightly more precise formula and is adjusted for generally active users. Therefore, we used the HUNT equation for the HRmax estimation instead of the Inbar equation. For estimating the performance level, (VO2) = 111.33 − 0.42 H, where H is the resting heart rate, as per Uth et al. [48].

## 3. Results

The descriptive statistics of 413 male cyclists’ anthropometric measurements were presented using the mean, standard deviation, maximum, minimum, range, and percentile distributions (h, 50th, and 95th) for four countries (India, China, Singapore, and Taiwan). The 413 male cyclists had a mean age of 32 years (SD 11.5 years). These cyclists had a mean riding experience of 5 years (SD 4 years). Table 1 presents the summary (mean, minimum, and maximum) of the cyclists’ characteristics from the four countries. Table A3 in Appendix A presents the individual country’s cyclist characteristics. Table 2 and Table A1 present the descriptive statistics for 18 anthropometric measurements (including BMI) among the four countries.

The Kruskal–Wallis test showed (Table 3) a statistically significant difference in the anthropometric measurements among these countries. The Kruskal–Wallis test’s H value is presented (Table 3), which indicates a 5% probability of summarizing that a difference presents when there is no actual difference. The mean Kruskal–Wallis test rank is shown (in Table 3), where the average of the ranks for all the anthropometric observations within each sample of the countries is displayed.

The Mann–Whitney U test results are summarized in Table 4 and Table A2. From the results in Table 4, the comparative analysis between the Singaporean and Taiwanese cyclists indicated that there is a significant difference (*p* < 0.05) among all anthropometric measurements, except for body weight. By comparing the median of the body weights between the Singaporeans and Taiwanese (see Table A2), the Mann–Whitney U test indicated that the cyclist’s weight was greater for Taiwanese (Mdn = 109.83) than for the Singaporeans (Mdn = 96.8). The comparative analysis between the Singaporean and Chinese cyclists indicates that there is a significant difference (*p* < 0.05) among all anthropometric measurements, except for foot breadth and BMI. Nevertheless, the test results indicate that the BMI of cyclists was greater for the Chinese (Mdn = 106.87) than for the Singaporeans (Mdn = 96.98), U = 4648, *p* = 0.23. In a comparison of the Singaporean cyclists with Indian cyclists, mostly the sitting-related anthropometric measurements (stature body height, shoulder height (sitting), elbow-to-elbow breadth, foot breadth, and BMI) were insignificant (*p* > 0.009) between the two counties. However, other anthropometric measurements were found to be significantly different (*p* < 0.05) from each other. The comparative analysis between the Taiwanese and Chinese cyclists indicated that there is a significant difference (*p* < 0.05) among most of the anthropometric measurements, except for weight, stature, elbow height (sitting), and lower leg length. The comparative analysis between the Indian and Taiwanese cyclists indicated that there is a significant difference (*p* < 0.05) among all anthropometric measurements, except for weight, buttock extension, and hand length. The test results indicated that the cyclists’ weight was greater for Taiwan (Mdn = 109.42) than India (Mdn = 101.65), U = 5104, *p* = 0.35. The results comparison between the Indian and Chinese cyclists indicated that there is a significant difference (*p* < 0.05) among most of the anthropometric measurements, except for acromion-grip length, foot breadth, BMI, and weight.

## 4. Discussion

This study collected anthropometric data from four different populations (Indian, Singaporean, Taiwanese, and Chinese). The body dimensions were summarized. The presented percentile values can be applied as a guide for product design, especially in the sitting-related activities (e.g., cycling) among the four groups, in general. According to the statistical results, most of the body dimensions were significantly different among the selected ethnic populations.

Table 2 shows the descriptive statistics for the 17 body dimensions for the Indian, Singaporean, Taiwanese, and Chinese populations. Among the four Asian populations, the Chinese males had the highest stature, while the Singaporean males presented the shortest stature. For the Taiwanese males, the smallest body dimensions were obtained on shoulder height (sitting), shoulder-elbow length, acromion-grip length, elbow-hand length, buttock-popliteal length, buttock-knee length, and elbow-to-elbow breadth when compared with the other three populations. Moreover, the five largest (lower leg length, knee height, elbow-hand length, buttock-popliteal length, and buttock-knee length) and three most minor (buttock extension, elbow height (sitting), hand length) body dimensions were found in the Indian population compared to the others. The Singaporeans had the greatest buttock extension and acromion-grip length but the smallest lower leg length, knee height, hip breadth (sitting), and foot breadth. The Chinese males presented with the most significant body size for shoulder height (sitting), shoulder-elbow length, hand length, and body weight among the four Asian populations. The differences in the body dimensions among the four Asian populations can be contributed to geographical factors, such as ethnicity, nutrition, economic development, and lifestyle [21,29]. These results support the previous studies [27,33] that reported on the geographic factors influencing genetic differentiation in ethnic populations, especially for stature and body weight. The geographical condition was related to unique socioeconomic statuses, activity, and nutrition intake and generated the different levels of medical and social services, which impacted the differences in body dimensions [21]. The mentioned specific body characteristics between the four populations should be considered as valuable information for bicycle designers to adequately satisfy the differences in overseas customers. For example, when designing a bike to be imported to Singapore, a redesign process is needed for a better fitting based on the Singaporean males’ specific body dimensions (e.g., a shorter lower leg length and knee height).

While comparing Singaporean, Indian, Chinese, and Taiwanese cyclists’ anthropometric measurements, there is a significant difference. Specifically, the Taiwanese cyclists’ weight is greater than that of Singaporeans. The Chinese cyclists’ BMI is greater than that of Singaporeans. Perhaps, these differences might be due to the different food diets, years of practice, and so on. In a comparison of the Singaporean cyclists with Indian cyclists, mostly sitting-related anthropometric measurements were insignificantly different from each other. Regarding the comparison of the Taiwanese cyclists with cyclists from other countries (China/India), the anthropometric measurements were significantly different from each other. However, a few of the anthropometric measurements (weight, stature, elbow height (sitting), lower leg length, buttock extension, and hand length) were insignificantly different from each other. Perhaps, these could be the same ethnicity or migration of cyclists from their native country.

Anthropometric data vary based on many factors, such as age and gender [27,31,32]. In the current study, we selected similar age groups among the four populations and limited the study to male subjects to avoid other influences. Apart from the geographical differences, it should be noted that there are cultural differences between the four populations. Many pieces of research focused on the body morphological differences among various ethnic populations. Moreover, lifestyle, occupation, genetics, social environment, labor structure, and economic levels play an important role in affecting the anthropometric body measurements of a population group [29]. For a global marketing business, it is essential for product designers to consider the anthropometric differences of nations in the design process. When realizing the differences between populations and applying them to a product design, the products are then designed in accordance with the user’s body characteristic requirements.

The descriptive statistical outcomes could be applied to bicycle design. For instance, the 95th percentile of weight can be used in the bicycle’s seat design. This application could facilitate the weight-carrying capacity of a bicycle seat for up to 95% of the bicyclists. Similarly, the 50th percentile value applications in bicycle design would be facilitated to cover 50% of the bicyclist population. In particular, range (5th to 95th percentile) values could be applied in the seat-height adjustment system to cover 95% of the bicyclist population. 

A validation was applied to evaluate the margin of errors in the selected body dimensions among the four populations. Across all data for the four populations, the %TEM test results reported that the intra- and inter-reliability of the current study was 0.15% to 1.73% and 0.11% to 1.57%, respectively. Based on the study of Arunachalam et al. [28], this research agreed that the %TEMs of an intra-/inter- less than 2% were considered to be highly reliable. Hence, our results suggested that anthropometry (i.e., anthropometrists-1, measurement protocol, instrument) is a trustworthy method for further data collection and comparisons. Meanwhile, all measurements in the current study were performed with rational precision and reliability during the collection of the body dimensions and were verified.

As per the literature survey, there was no any comparative investigation performed for the cyclists’ anthropometric measurements in these countries (Singapore, India, Taiwan, and China). The current investigative study is a first-of-its-kind to carry out this approach. Overall, the current study’s findings, from the qualitative analyses, could lead to performing large-scale anthropometric surveys by researchers and ergonomists. Further, an individual country’s anthropometric database for cyclists could be developed to improve the betterment of bicycle designs.

The current study considered only males and the age groups between 18 to 60. Considering both female and male cyclists, the male population was supposed to be greater. Thus, this study was conducted with male cyclists. However, a similar line of study has been planned for females from these countries in the future. Due to limited resources, a comparative analysis was performed for these countries (Singapore, India, Taiwan, and China) only. Due to the time constraints, the sample sizes were marginally small, which arose as a limitation in the parametric statistical analyses to generalize the results. However, the study sample size matches the minimal sample size. A large-sample study may include each country’s ethnicity, etc., which may also affect the cyclist’s anthropometric measurements.

## 5. Conclusions

This study was conducted to characterize male cyclists from India, Taiwan, China, and Singapore through an anthropometric study. Seventeen body dimensions were studied using anthropometric kits. Based on the statistical analysis, it has been established that most of the standing anthropometric measurements were different from each other. However, the weight and sitting-related anthropometrics did not differ much. This is the first study of its kind to present a descriptive analysis of four different countries’ (Singapore, India, Taiwan, and China) anthropometric measurements for cyclists. The involvement of the percentile values in bicycle design will improve the ergonomic benefits for the bicyclist. Therefore, this study acts as a dataset for performing bicycle ergonomic design in these countries (Singapore, India, Taiwan, and China).

## Figures and Tables

**Table 1:** Summary of cyclists’ characteristics (*n* = 406).

Characteristic	Mean	Range	
		Min	Max
Age (Yrs.)	28	18	50
Body surface area (m^2^)	1.79	1.47	2.20
HRmax (beats/min)	193	179	199
Performance level (VO2)	79	78	81
Years of practice	10	1	32
Weekly training load (km)	2664	2162	3033

**Table 2:** Descriptive statistics of cyclists’ anthropometric measurements. Note: All measurements are in mm unless specified.

S. No	Anthropometric	Countries	Median	Mean	Std. Deviation	Minimum	Maximum	Interquartile Range	Range	Percentiles		
										5th	50th	95th
1	Stature body height	India	1680	1694.18	68.78	1540.00	1875.00	99.25	335.00	1599.00	1680.00	1823.75
		Singapore	1677	1683.63	76.20	1521.00	1900.00	102.75	379.00	1552.40	1677.00	1820.00
		Taiwan	1715.8	1720.97	54.95	1587.40	1855.20	83.52	267.80	1634.16	1715.80	1813.36
		China	1720	1722.42	59.78	1600.00	1933.40	80	333.40	1630.00	1720.00	1818.70
2	Buttock extension	India	855	844.39	60.43	575.00	980.00	70	405.00	725.00	855.00	935.00
		Singapore	971	966.27	48.90	820.00	1073.00	57	253.00	877.10	971.00	1039.95
		Taiwan	859.35	862.21	39.63	775.00	955.00	55.92	180.00	797.28	859.35	930.62
		China	891	887.83	78.10	470.00	1099.30	74	629.30	775.10	891.00	1000.00
3	Shoulder height (Sitting)	India	572.2	577.22	30.92	507.10	654.00	44.48	146.90	522.38	572.20	632.60
		Singapore	569.5	579.71	46.84	493.00	711.00	54	218.00	512.10	569.50	653.95
		Taiwan	515.6	516.80	35.62	435.30	599.30	53.6	164.00	460.91	515.60	580.50
		China	622.5	621.94	43.38	450.00	740.00	44.5	290.00	555.26	622.50	700.00
4	Shoulder-elbow length	India	352.85	354.44	22.38	301.70	410.10	27.52	108.40	315.85	352.85	393.53
		Singapore	317.5	336.00	52.66	263.00	432.00	32.75	169.00	268.15	317.50	419.95
		Taiwan	253.85	254.81	18.63	210.00	312.10	24.92	102.10	222.52	253.85	285.33
		China	358	368.06	37.35	310.00	500.00	33	190.00	323.14	358.00	450.00
5	Elbow height (Sitting)	India	224.2	222.25	27.01	160.60	284.90	39.25	124.30	175.38	224.20	270.93
		Singapore	243	243.71	31.81	190.00	358.00	45.5	168.00	198.00	243.00	292.95
		Taiwan	261.05	262.00	27.90	201.30	321.80	43.85	120.50	219.68	261.05	311.06
		China	257	251.54	39.03	140.00	320.00	53	180.00	179.20	257.00	309.78
6	Lower leg length	India	444.6	447.60	41.19	373.80	710.30	50.3	336.50	393.05	444.60	498.05
		Singapore	410	413.90	27.88	356.00	493.00	35.5	137.00	370.15	410.00	464.75
		Taiwan	417.3	419.97	26.62	362.60	477.00	34.6	114.40	375.66	417.30	465.67
		China	428.5	425.69	40.55	160.00	500.00	40	340.00	366.94	428.50	480.00
7	Knee height	India	551.6	551.23	44.03	463.10	835.40	47.5	372.30	492.00	551.60	603.63
		Singapore	491.5	501.20	39.74	425.00	680.00	53.75	255.00	453.25	491.50	572.95
		Taiwan	506.85	508.14	24.76	458.60	560.10	38.57	101.50	469.15	506.85	549.68
		China	534	532.95	28.04	430.00	601.00	35.1	171.00	490.00	534.00	590.20
8	Acromion-grip length	India	627.55	629.17	41.14	528.60	750.00	51.2	221.40	561.85	627.55	702.58
		Singapore	650	651.90	60.04	542.00	800.00	54	258.00	553.70	650.00	764.75
		Taiwan	566.825	568.96	27.26	491.50	635.80	33.67	144.30	521.44	566.83	617.38
		China	625	630.97	40.45	550.00	750.00	52	200.00	580.00	625.00	716.00
9	Hand length	India	177.95	177.63	13.38	150.90	209.30	21.58	58.40	155.60	177.95	199.80
		Singapore	183	182.17	8.47	160.00	205.00	11	45.00	168.00	183.00	196.95
		Taiwan	178.1	178.01	9.98	148.00	200.50	10.97	52.50	159.38	178.10	195.49
		China	185	185.40	21.83	76.00	300.00	15.9	224.00	170.00	185.00	209.20
10	Elbow-hand length	India	475.05	474.55	25.91	420.30	538.90	35.65	118.60	433.50	475.05	523.68
		Singapore	461.5	462.06	26.58	386.00	520.00	36.75	134.00	413.10	461.50	497.00
		Taiwan	367.15	366.57	19.59	317.00	420.60	24.43	103.60	340.24	367.15	408.84
		China	450	448.96	28.26	270.00	500.00	29	230.00	406.80	450.00	490.00
11	Buttock-popliteal length	India	497.95	497.73	39.29	398.30	569.80	57.82	171.50	436.25	497.95	563.18
		Singapore	458	455.22	26.96	356.00	508.00	29.75	152.00	400.15	458.00	497.00
		Taiwan	353.05	354.83	24.88	289.30	416.00	33.55	126.70	307.97	353.05	398.92
		China	440	438.40	38.95	330.00	524.00	41.5	194.00	360.00	440.00	508.00
12	Buttock-knee length	India	596.65	595.15	35.29	497.70	675.40	51.4	177.70	538.95	596.65	655.03
		Singapore	568.5	562.25	29.21	470.00	624.00	32.5	154.00	491.70	568.50	596.85
		Taiwan	455	453.76	31.04	351.70	517.10	40.67	165.40	393.27	455.00	499.90
		China	549.4	547.53	68.97	478.56	671.00	47.5	671.00	480.00	549.40	630.80
13	Elbow-to-elbow breadth	India	434	430.31	38.41	330.00	533.00	60	203.00	357.75	434.00	488.75
		Singapore	430.5	434.95	28.67	379.00	533.00	40.5	154.00	399.10	430.50	489.50
		Taiwan	401.55	399.11	23.15	349.20	449.80	35.65	100.60	361.62	401.55	435.73
		China	450	451.58	48.70	150.00	600.00	40	450.00	393.00	450.00	518.94
14	Hip breadth (Sitting)	India	340	336.88	27.54	270.00	425.00	34.75	155.00	290.00	340.00	380.00
		Singapore	306	307.17	21.19	250.00	371.00	26.75	121.00	273.00	306.00	347.75
		Taiwan	376.95	376.75	26.44	321.90	445.60	34.05	123.70	327.20	376.95	421.18
		China	360	360.11	38.76	250.00	550.00	40	300.00	299.20	360.00	413.54
15	Foot breadth	India	100	101.72	8.27	85.00	115.00	15	30.00	90.00	100.00	115.00
		Singapore	102	101.06	6.70	82.00	114.00	8	32.00	90.05	102.00	112.90
		Taiwan	104.05	104.31	4.70	91.60	120.30	6.13	28.70	96.65	104.05	111.90
		China	100	101.98	16.82	76.00	240.00	10.7	164.00	88.40	100.00	121.60
16	BMI (kg/m²)	India	23.81	23.80	3.67	13.71	31.74	5.71	18.03	18.48	23.81	29.25
		Singapore	22.8	23.27	2.13	18.94	31.63	2.83	12.69	19.96	22.80	27.22
		Taiwan	22.18	22.37	2.61	17.96	31.14	3.05	13.18	18.43	22.18	27.29
		China	23.53	23.71	3.22	17.04	34.84	3.53	17.80	18.62	23.53	29.26
17	Weight (kg)	India	68.5	68.24	10.72	38.00	96.00	16.13	58.00	51.00	68.50	83.50
		Singapore	64	65.94	7.42	50.00	95.00	9.75	45.00	58.00	64.00	79.95
		Taiwan	67.5	67.59	8.56	53.00	92.00	11.25	39.00	55.00	67.50	85.00
		China	70	70.36	10.72	52.00	110.00	12	58.00	55.00	70.00	90.80

**Table 3:** Results of the Kruskal–Wallis test.

Anthropometric Variables	Kruskal– Wallis Test	*p*-Value	Group’s Mean Rank			
	H	*p*	Singapore (SGD)	India (INR)	Taiwan (ROC)	China (PRC)
Stature body height	26.36	0.0001	169.98	183.48	235.73	237.13
Buttock extension	178.65	0.002	335.86	132.27	153.7	212.2
Shoulder height (Sitting)	199.9	0.002	219.33	216.13	82.43	314.01
Shoulder-elbow length	235.15	0.004	229.34	261.06	57.34	284.74
Elbow height (Sitting)	79.01	0.0001	199.94	126.52	266.14	234.25
Lower leg length	52.29	0.004	157.25	271.71	183.76	213.88
Knee height	117.67	0.003	133.29	289.69	157.2	246.33
Acromion-grip length	157.64	0.0001	271.79	238.24	83.55	239.6
Hand length	31.07	0.0001	221.73	178.21	175.48	254.21
Elbow-hand length	254.18	0.0001	262.53	297.87	55.19	217.57
Buttock-popliteal length	278.97	0.002	242.38	328.1	58.97	202.72
Buttock-knee length	259.74	0.0001	243.12	315	58.15	216.07
Elbow-to-elbow breadth	123.45	0.003	227.42	215.63	104.88	283.56
Hip breadth (Sitting)	206.55	0.0001	82.4	179.1	309.43	250.73
Foot breadth	19.91	0.004	192.96	207.82	247.73	177.9
BMI	18.14	0.0001	207.61	230.72	166.84	223.79
Weight	11.97	0.0001	176.82	216.51	201.43	232.43

**Table 4:** Results of Mann–Whitney U test.

**Statistical Parameters**	**SGD vs. ROC**		**SGD vs. PRC**		**SGD vs. INR**	
	**Mann–Whitney U**	**Sig (2-Tailed)** ***p*-Value**	**Mann–Whitney U**	**Sig (2-Tailed) *p*-Value**	**Mann–Whitney U**	**Sig (2-Tailed)** ***p*-Value**
BMI	4027	0.003	4648	0.23	4490	0.09
Weight	4629.5	0.11	3724	0.001	4278.5	0.02
Stature body height	3619.5	0	3517	0	4811.5	0.35
Buttock extension	576.5	0	1701	0	486.5	0
Shoulder height (Sitting)	1605.5	0	2662.5	0	5174.5	0.95
Shoulder-elbow length	402.5	0	3532	0	4154	0.01
Elbow height (Sitting)	3515.5	0	4251.5	0.03	3223	0
Lower leg length	4532.5	0.07	3680.5	0	2461.5	0
Knee height	4216	0.01	2318.5	0	1744.5	0
Acromion-grip length	1067.5	0	4053.5	0.009	4050	0.006
Hand length	3966.5	0.002	4179	0.02	4090	0.008
Elbow-hand length	36.5	0	3671	0	4010	0.005
Buttock-popliteal length	81	0	3591	0	1959.5	0
Buttock-knee length	102.5	0	4067	0.01	2531	0
Elbow-to-elbow breadth	1739.5	0	3372	0	4941	0.53
Hip breadth (Sitting)	206.5	0	1009	0	1974.5	0
Foot breadth	3792	0	4667.5	0.24	4821	0.36
**Statistical Parameters**	**ROC vs. PRC**		**INR vs. ROC**		**INR vs. PRC**	
	**Mann–Whitney U**	**Sig (2-Tailed)** ***p*-Value**	**Mann–Whitney U**	**Sig (2-Tailed) *p*-Value**	**Mann–Whitney U**	**Sig (2-Tailed)** ***p*-Value**
BMI	3968	0.001	4019	0.001	5092.5	0.54
Weight	4606	0.051	5104	0.35	5016	0.43
Stature body height	5376	0.849	4064.5	0.001	3968.5	0.001
Buttock extension	3705	0	4684.5	0.06	3125.5	0
Shoulder height (Sitting)	313.5	0	1148	0	1966.5	0
Shoulder-elbow length	1	0	4	0	4424.5	0.03
Elbow height (Sitting)	4786.5	0.124	1700.5	0	2775	0
Lower leg length	4640.5	0.061	3100	0	3777	0
Knee height	2707.5	0	1900.5	0	3823.5	0
Acromion-grip length	914	0	1204	0	5265	0.83
Hand length	3395.5	0	5456	0.89	3528	0
Elbow-hand length	142	0	1	0	2607	0
Buttock-popliteal length	492	0	7	0	1507.5	0
Buttock-knee length	385	0	5	0	2299.5	0
Elbow-to-elbow breadth	1029	0	2677.5	0	3678	0
Hip breadth (Sitting)	3635.5	0	1571	0	3169.5	0
Foot breadth	3515.5	0	4646.5	0.04	4784.5	0.18

Note: Singaporean (SGD); Taiwanese (ROC); Chinese (PRC); Indian (INR).

## Data Availability

The datasets generated during and/or analyzed during the current study are available from the corresponding author on reasonable request.

## Appendix A

**Table A1:** Additional descriptive statistics. Note: All measurements are in mm unless specified.

Anthropometric Variables	Confidence Interval		Chinese	Indian	Taiwanese	Singaporeans
Stature body height	95% Confidence Interval for Mean	Lower Bound	1710.74	1680.81	1710.38	1668.51
		Upper Bound	1734.10	1707.56	1731.55	1698.75
	Std. Deviation		59.78	68.78	54.95	76.20
Buttock extension	95% Confidence Interval for Mean	Lower Bound	872.56	832.64	854.58	956.57
		Upper Bound	903.09	856.15	869.84	975.97
	Std. Deviation		78.10	60.43	39.63	48.90
Shoulder height (Sitting)	95% Confidence Interval for Mean	Lower Bound	613.47	571.20	509.94	570.42
		Upper Bound	630.42	583.23	523.66	589.00
	Std. Deviation		43.38	30.92	35.62	46.84
Shoulder-elbow length	95% Confidence Interval for Mean	Lower Bound	360.76	350.09	251.22	325.55
		Upper Bound	375.36	358.79	258.40	346.45
	Std. Deviation		37.35	22.38	18.63	52.66
Elbow height (Sitting)	95% Confidence Interval for Mean	Lower Bound	243.91	217.00	256.62	237.40
		Upper Bound	259.17	227.50	267.37	250.02
	Std. Deviation		39.03	27.01	27.90	31.81
Lower leg length	Mean		425.69	447.60	419.97	413.90
	95% Confidence Interval for Mean	Lower Bound	417.77	439.58	414.84	408.37
		Upper Bound	433.62	455.61	425.10	419.43
	Std. Deviation		40.55	41.19	26.62	27.88
Knee height	95% Confidence Interval for Mean	Lower Bound	527.47	542.67	503.37	493.31
		Upper Bound	538.43	559.79	512.91	509.09
	Std. Deviation		28.04	44.03	24.76	39.74
Acromion-grip length	95% Confidence Interval for Mean	Lower Bound	623.07	621.17	563.71	639.99
		Upper Bound	638.88	637.18	574.21	663.81
	Std. Deviation		40.45	41.14	27.26	60.04
Hand length	95% Confidence Interval for Mean	Lower Bound	181.13	175.03	176.09	180.49
		Upper Bound	189.67	180.23	179.93	183.85
	Std. Deviation		21.83	13.38	9.98	8.47
Elbow-hand length	95% Confidence Interval for Mean	Lower Bound	443.44	469.51	362.80	456.79
		Upper Bound	454.49	479.59	370.34	467.33
	Std. Deviation		28.26	25.91	19.59	26.58
Buttock-popliteal length	95% Confidence Interval for Mean	Lower Bound	430.79	490.09	350.04	449.87
		Upper Bound	446.01	505.37	359.62	460.57
	Std. Deviation		38.95	39.29	24.88	26.96
Buttock-knee length	95% Confidence Interval for Mean	Lower Bound	534.05	588.29	447.78	556.45
		Upper Bound	561.01	602.02	459.74	568.05
	Std. Deviation		68.97	35.29	31.04	29.21
Elbow-Elbow breadth	95% Confidence Interval for Mean	Lower Bound	442.06	422.84	394.65	429.26
		Upper Bound	461.10	437.78	403.56	440.64
	Std. Deviation		48.70	38.41	23.15	28.67
Hip breadth (Sitting)	95% Confidence Interval for Mean	Lower Bound	352.53	331.53	371.65	302.97
		Upper Bound	367.68	342.24	381.84	311.37
	Std. Deviation		38.76	27.54	26.44	21.19
Foot breadth	95% Confidence Interval for Mean	Lower Bound	98.69	100.11	103.40	99.73
		Upper Bound	105.27	103.33	105.21	102.39
	Std. Deviation		16.82	8.27	4.70	6.70
Weight (kg)	95% Confidence Interval for Mean	Lower Bound	68.26	66.15	65.94	64.47
		Upper Bound	72.45	70.32	69.24	67.41
	Std. Deviation		10.72	10.72	8.56	7.42
BMI (kg/m^2^)	95% Confidence Interval for Mean	Lower Bound	23.08	23.09	21.87	22.84
		Upper Bound	24.33	24.51	22.87	23.69
	Std. Deviation		3.22	3.67	2.61	2.13

**Table A2:** Mean rank of Mann–Whitney test.

Anthropometric Variables	Singaporean vs. Taiwanese	Singaporean vs. Chinese	Singaporean vs. Indian	Taiwanese vs. Chinese	Indian vs. Taiwanese	Indian vs. Chinese
Stature body height	86.7	85.67	98.62	104.22	91.58	90.66
	119.35	117.85	106.24	105.81	119.16	117.47
Buttock extension	150.74	136.49	149.64	88.45	97.54	82.55
	58.94	68.51	57.18	122.03	113.31	125.66
Shoulder height (Sitting)	140.45	77.13	102.76	56.46	147.46	71.41
	68.65	126.15	102.25	154.96	64.33	136.91
Shoulder-elbow length	152.48	85.82	92.04	53.51	158.46	95.04
	57.3	117.71	112.56	157.99	53.54	113.04
Elbow height (Sitting)	85.66	93.02	122.27	111.34	68.85	79.18
	120.33	110.72	83.49	98.47	141.46	129.06
Lower leg length	95.83	87.31	75.12	97.28	128.69	119.18
	110.74	116.27	128.83	112.95	82.75	88.67
Knee height	92.66	73.69	67.95	79.04	140.23	118.74
	113.73	129.49	135.73	131.71	71.43	89.12
Acromion-grip length	145.83	112.97	114	62.12	146.92	104.88
	63.57	91.35	91.44	149.13	64.86	103.12
Hand length	116.84	92.29	113.6	85.53	104.96	86.42
	90.92	111.43	91.83	125.03	106.03	121.75
Elbow-hand length	156.14	116.79	90.6	54.84	158.49	130.43
	53.84	87.64	113.94	156.62	53.51	77.31
Buttock-popliteal length	155.69	117.59	70.1	58.14	158.43	141
	54.26	86.86	133.66	153.22	53.57	66.64
Buttock-knee length	155.48	112.83	75.81	57.13	158.45	133.39
	54.47	91.49	128.16	154.26	53.55	74.33
Elbow-to-elbow breadth	139.11	84.22	105.09	63.21	132.75	87.87
	69.91	119.26	100.01	148.01	78.76	120.29
Hip breadth (Sitting)	52.57	60.59	70.25	122.2	67.61	82.98
	151.55	142.2	133.51	87.3	142.68	125.23
Foot breadth	88.42	106.83	98.71	123.33	97.18	109.5
	117.73	97.32	106.14	86.13	113.67	98.45
BMI	116.23	96.98	95.4	90.93	119.86	106.53
	91.49	106.87	109.33	119.48	91.42	101.44
Weight	96.8	87.74	93.29	96.95	109.42	100.73
	109.83	115.84	111.36	113.28	101.65	107.3

**Table A3:** Summary of cyclists’ characteristics (*n* = 406).

Characteristic	Singapore (SGD)			India (INR)			Taiwan (ROC)			China (PRC)		
	Mean	Range		Mean	Range		Mean	Range		Mean	Range	
		Min	Max		Min	Max		Min	Max		Min	Max
Age (Yrs.)	27	20	56	25	18	37	30	20	49	32	19	59
Body surface area (m^2^)	1.76	1.46	2.20	1.78	1.33	2.11	1.79	1.54	2.33	1.83	1.33	2.33
HRmax (beats/min)	194	175	198	195	187	199	192	180	198	191	173	199
Performance level (VO2)	79	78	82	79	78	80	79	78	81	79	78	82
Years of practice	9	2	38	7	1	19	12	2	31	14	1	41
Weekly training load (km)	2650	2452	2978	2785	2125	3250	2700	1985	2985	2520	2085	2920

Note: The body surface area has been estimated based on the Fujimoto and Watanabe (1969) formula; HR_max_ (beats/min) = [211 − 0.64 × Age]; performance level (VO2) = 111.33 − 0.42 H, where H is resting heart rate, as per Uth et al. [48].

**Table A4:** Technical Error of Measurement for 16 Anthropometrics (*n* = 10).

Anthropometric Variables	Intra-Observer Technical Error (%TEM)				Inter-Observer Technical Error (%TEM)			
	Singapore (SGD)	India (INR)	Taiwan (ROC)	China (PRC)	Singapore (SGD)	India (INR)	Taiwan (ROC)	China (PRC)
Stature body height	0.47	0.51	0.35	0.49	0.11	0.24	0.34	0.45
Buttock extension	0.32	0.34	0.28	0.30	0.35	0.48	0.54	0.54
Shoulder height (Sitting)	0.94	0.93	0.86	0.84	1.20	1.09	1.24	0.59
Shoulder-elbow length	1.11	1.24	0.92	1.01	0.98	1.09	1.32	1.45
Elbow height (Sitting)	1.73	1.68	1.45	1.64	1.20	1.54	1.05	0.95
Lower leg length	1.2	1.23	0.89	0.98	0.89	0.97	0.85	1.57
Knee height	0.40	0.54	0.65	0.35	0.68	0.78	0.56	1.25
Acromion-grip length	0.64	0.79	0.68	0.54	0.60	0.63	0.70	1.01
Hand length	1.33	1.35	1.21	1.01	1.21	1.51	1.24	1.32
Elbow-hand length	0.91	0.93	0.78	0.89	0.78	0.88	0.78	0.85
Buttock-popliteal length	1.10	1.39	1.56	1.23	0.68	0.87	0.74	0.65
Buttock-knee length	0.86	0.66	0.98	0.78	0.65	0.73	0.69	0.95
Elbow-to-elbow breadth	0.64	0.80	0.75	0.74	0.85	0.89	0.94	1.02
Hip breadth (Sitting)	0.95	0.96	0.98	0.9	1.01	1.18	1.25	1.35
Foot breadth	0.64	0.78	0.85	0.54	0.94	0.98	0.86	0.89
Weight	0.23	0.15	0.3	0.25	0.23	0.34	0.32	0.35
